# Eosinophil Recruitment and Activity in CNS Lesions of MOG Antibody–Associated Myelitis Following Influenza Vaccination: A Case Report

**DOI:** 10.1155/crnm/6886402

**Published:** 2026-05-24

**Authors:** Hiroshi Adachi, Misa Takashima, Naonobu Futamura

**Affiliations:** ^1^ Department of Neurology, National Hospital Organization Hyogo Chuo National Hospital, Hyogo, Japan; ^2^ Department of Gastroenterology, Kobe Rosai Hospital, Hyogo, Japan, rofuku.go.jp

**Keywords:** central nervous system (CNS), cerebrospinal fluid (CSF), eosinophil, influenza vaccination, myelin oligodendrocyte glycoprotein (MOG)

## Abstract

The appearance of eosinophils in the cerebrospinal fluid (CSF) is considered atypical in patients with myelin oligodendrocyte glycoprotein (MOG) antibody–associated disease (MOGAD). In this article, we report the case of a 24‐year‐old male who received an influenza vaccine and subsequently presented with sensory disturbances in his limbs and trunk, accompanied by urinary retention. On spinal cord magnetic resonance imaging (MRI), T2‐weighted images showed intramedullary high‐intensity lesions. Meanwhile, brain MRI revealed no lesions. CSF testing showed pleocytosis (180 cells/μL), and eosinophils were present in approximately 6% of the CSF white blood cells. Additionally, MOG antibodies (MOG‐IgG) were present in the serum and CSF, leading to the diagnosis of MOG‐IgG–associated myelitis induced by immunization following influenza vaccination. It is speculated that perivascular infiltration of eosinophils can occur within demyelinating lesions in patients with MOGAD, possibly due to elevated levels of eosinophil‐associated cytokines and chemokines in the central nervous system (CNS) lesions. However, it remains unclear whether immunological responses triggered by vaccination contribute to the secretion of these mediators in such lesions.

## 1. Introduction

Eosinophils in the cerebrospinal fluid (CSF) are uncommon although they are sometimes observed in patients with parasitic infections, allergic diseases, Hodgkin lymphoma, fungal infections, or ventriculoperitoneal (VP) shunts. Moreover, in occasional cases of neuromyelitis optica spectrum disorder (NMOSD), eosinophils are observed in the CSF, in contrast to multiple sclerosis (MS), in which they are generally absent [1, 2].

It is widely recognized that the presence of eosinophils in the CSF reflects infiltration of eosinophils in the central nervous system (CNS) following disruption of the blood–brain barrier (BBB). Specifically, pathogenic mechanisms in NMOSD are that aquaporin‐4 (AQP4) antibodies (AQP4‐IgG) bind to AQP4 after increased permeability of the BBB. Complement activation resulting from the antigen–antibody interaction, along with cytokine production induced by astrocytic injury, causes migration of lymphocytes, neutrophils, and eosinophils from peripheral blood to the CNS. The activated eosinophils release eosinophil cationic protein (ECP) during degranulation, which exerts cytotoxicity. Thus, the eosinophil recruitment and function have been progressively elucidated in NMOSD [3, 4].

By contrast, the infiltration of eosinophils may not be a typical feature of myelin oligodendrocyte glycoprotein (MOG) antibody–associated disease (MOGAD) [5], and previous reports regarding CSF eosinophils in MOGAD are limited in the literature [6–9]. Pathologically, it is postulated that via the damaged BBB following nuclear factor kappa B (NF‐κB) signaling and increased oxidative stress [10], MOG antibodies (MOG‐IgG) penetrate from peripheral blood to the CNS and bind to MOG. In the CNS, the antigen–antibody interaction results in infiltration of lymphocytes, macrophages, and granulocytes possibly including eosinophils [11]. However, many aspects of the detailed pathology of MOGAD, including the role of eosinophils, are not elucidated.

Herein, we describe the case of a 24‐year‐old male who developed myelitis with MOG‐IgG positivity, characterized by the appearance of eosinophils in the CSF. The myelitis was likely induced by immunization following influenza vaccination. This unusual pathology involving eosinophils in the CSF of our patient warrants discussion to provide new clues to eosinophil recruitment and activity within CNS lesions in MOGAD, particularly in comparison with NMOSD.

## 2. Case Presentation

A 24‐year‐old Vietnamese male visited our hospital complaining of abnormal sensations in his limbs and trunk, accompanied by urinary retention. The patient had no relevant medical history except for an allergy to pollen, and he was not exposed to pollen at that time. He came to Japan 2 years ago and had not traveled overseas since then. He did not have a habit of eating raw fish or meat. Moreover, the patient did not have pets.

He received an unadjuvanted split inactivated vaccine against seasonal influenza 9 days before disease onset. At onset, he noticed paresthesia of his hands and lower limbs in parallel with dullness of temperature and pain sensation below the Th6 dermatome level. These sensory disturbances were dominant on the right side. Urinary retention was observed 14 days after the onset. He presented to our department 18 days after the onset and was subsequently admitted.

On admission, the patient presented with slight fever and mild headache without a skin rash. He had no neuropsychological symptoms and no cranial nerve palsies, including optic nerve disorders. He had no motor disturbances. In addition to the aforementioned abnormal sensations, deep sensations were mildly impaired in both distal legs. With regard to the pyramidal tract signs, patellar tendon reflexes were mildly brisk on both sides, whereas Babinski and Chaddock signs were absent.

**FIGURE 1:**
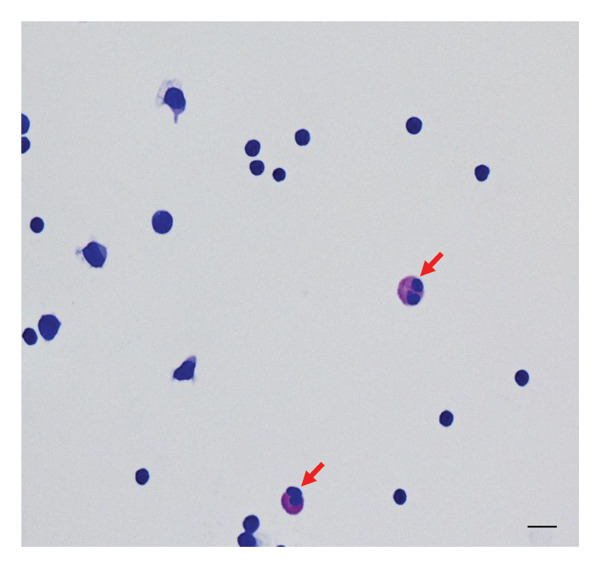
Cerebrospinal fluid (CSF) cytology using Giemsa stain. Eosinophils (indicated with arrows) and lymphocytes were observed at the initial lumber puncture before steroid pulse therapy. Scale bar: 10 μm.

**FIGURE 2:**
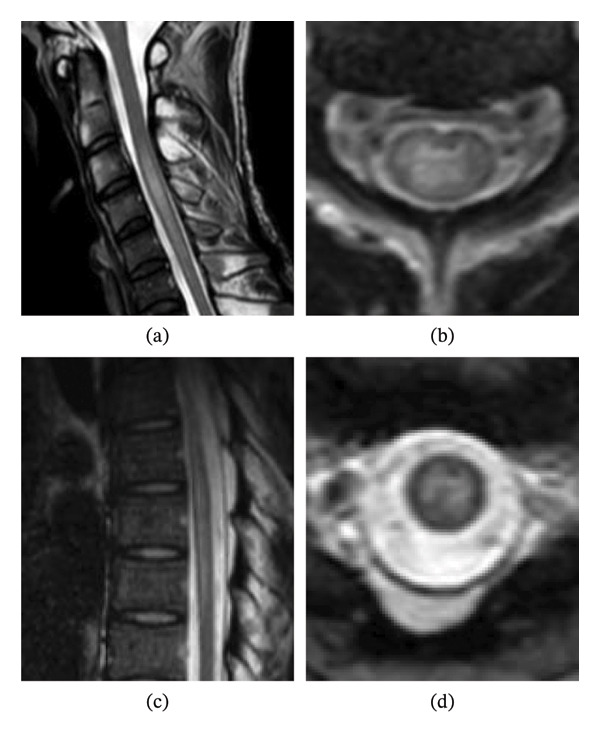
Magnetic resonance imaging (MRI) of spinal cord. T2‐weighted images showed intramedullary high‐intensity lesions with swelling, at C3–C5 (a, b) and Th6–Th8 (c, d) spinal levels ((a, c) sagittal views; (b, d) axial views).

Laboratory tests revealed normal white blood cell counts (4800/μL), including serum eosinophils (5.7%, 274/μL). Serum immunoglobulin E (IgE) levels were also normal. The patient tested negative for serum AQP4‐IgG. Serum antibody tests for collagen disease, vasculitis, viral infection, and syphilis were negative. First CSF testing before therapy revealed pleocytosis (180 cells/μL), and most of the cells were lymphocytes. Meanwhile, approximately 6% of CSF white blood cells were eosinophils, identified by cytology using Giemsa staining (Figure 1). The CSF total protein concentration was normal, and myelin basic protein levels (272.2 pg/mL) were elevated. No oligoclonal bands were observed. CSF adenosine deaminase activity (ADA) levels were normal. We also examined serum and CSF antibodies for parasitic infections, and those of *Toxocara canis*, *Ascaris suum*, and *Gnathostoma* were negative. On spinal cord magnetic resonance imaging (MRI), T2‐weighted images showed intramedullary high‐intensity lesions in the spinal cord, C3–C5, and Th6–Th8 segments (Figure 2). Brain MRI revealed no lesions.

He was diagnosed with acute myelitis, and intravenous methylprednisolone (IVMP) at 1 g/day was administered for 3 days per course. After two courses of IVMP, the patient’s symptoms completely disappeared. In follow‐up CSF analysis two weeks after the first testing, pleocytosis improved (15 cells/μL) and eosinophils were almost undetectable (less than 1% of CSF white blood cells). Oral prednisolone (15 mg/day) was initiated to prevent recurrence, and the patient was discharged.

After discharge, MOG‐IgG turned out to be positive by cell‐based assays in both serum (a titer of 1:256) and CSF (a titer of 1:1) samples collected on admission. The patient was finally diagnosed with MOG‐IgG–associated myelitis following influenza vaccination. Eight months after the onset, no relapse was observed, and oral prednisolone was discontinued following a taper.

## 3. Discussion

In the present case, the patient received an influenza vaccine and subsequently developed MOG‐IgG–associated myelitis characterized by the appearance of eosinophils in the CSF.

CSF eosinophils are sometimes observed in meningomyelitis associated with parasitic infections, while they are observed in only a few patients with inflammatory CNS demyelinating diseases. Jarius et al. [1] reported that CSF eosinophils were present in 10% of 117 samples from juvenile and adult patients with AQP4‐IgG–positive NMOSD. Previous histopathological studies have illustrated infiltration of eosinophils in addition to lymphocytes and macrophages in early active demyelinating NMOSD lesions [12, 13].

From an immunological perspective in NMOSD, infiltration of eosinophils is mediated by CCR3, a receptor for eotaxins which attract eosinophils. Correale and Fiol [14] demonstrated that CSF levels of eotaxin‐2 (CCL24) and eotaxin‐3 (CCL26), together with ECP, are elevated in patients with NMOSD. Therefore, this result indicates that eosinophils play a role in neuronal toxicity through eosinophil activation and degranulation in NMOSD.

By contrast, the presence of eosinophils in the CSF of MOG‐IgG–positive patients is considered rare. Recently, Jarius et al. [6] reported that eosinophils were present during acute attacks in 2.6% of 77 CSF samples from adult patients with MOGAD. On the other hand, among pediatric patients with MOGAD, CSF eosinophils were found during acute attacks in 6.7% of 45 samples [7]. Interestingly, in another recent study, CSF eosinophils were detected in 12 of 36 pediatric patients (33%) with MOGAD, which is higher than the results of other cohort studies for undetermined reasons [8]. The authors showed that the mean CSF eosinophil count was 3%, ranging from 1% to 18% of the CSF white blood cells. These findings indicate that eosinophils can infiltrate CNS lesions in patients with MOGAD, as well as in those with AQP4‐IgG–positive NMOSD.

With regard to histopathological assessments of CNS lesions in MOGAD, Takai et al. [15] demonstrated that the histopathological features exhibited a perivenous demyelination pattern as seen in acute disseminated encephalomyelitis (ADEM). In the perivascular lesions, infiltration of inflammatory cells (macrophages, T cells, and fewer B cells) was seen, whereas deposition of complement components and immunoglobulins, identified as humoral immune reactions, was less frequently observed in patients with MOGAD than in those with NMOSD. In another paper, Hoftberger et al. [16] illustrated a mixed pattern of both perivenous and confluent demyelination in brain biopsies from some patients with MOGAD. The lesions contained perivascular and parenchymal infiltration not only of lymphocytes and neutrophils but also of eosinophils, along with complement deposition. Taken together, these assessments suggest that MOGAD exhibits different pathological patterns, and the degree of eosinophil infiltration may vary depending on the pathological patterns.

In terms of CSF cytokines and chemokines in MOGAD, Kaneko et al. [17] reported that this disease typically showed upregulation of several T helper type 1 (Th1)–related and regulatory T cell–related cytokines in addition to Th17‐related cytokines such as interleukin‐6 (IL‐6). When focusing on eosinophil‐associated cytokines and chemokines, CSF levels of IL‐5, a Th2‐related key cytokine involved in eosinophil activation, are mildly elevated in a subset of patients [17], which is possibly correlated with increased CSF levels of eotaxins [14]. It is suggested that among these patients, greater numbers of eosinophils are recruited into the CNS and play an important pathogenic role in the development of demyelination. The CSF levels of these cytokines and chemokines may be increased depending on specific triggers of acute attacks or particular background factors in patients with MOGAD.

Notably, in the present case, MOG‐IgG–associated myelitis developed after influenza vaccination. The vaccination is generally considered safe and well tolerated. Despite its benefits, it is known that influenza vaccination very rarely causes CNS demyelination (e.g., ADEM) following immunization [18], and previous reports of MOGAD after influenza vaccination are still extremely limited in the literature [17, 19]. In our patient, immunization following influenza vaccination was considered to be a key trigger for MOG‐IgG positivity in serum and CSF. Pathologically, it has been proposed that, given the nature of the split‐product influenza vaccines, their antigens are not primarily recognized by Toll‐like receptors (TLRs). Instead, the antigen recognition is mediated via B cell receptor (BCR) signaling, leading to activation and differentiation of MOG‐specific B cells through interactions with T cells and the subsequent production of MOG‐IgG. The split inactivated influenza vaccines do not contain adjuvants, and as a result, Th2‐dominant responses tend to be promoted in the immunological process [20]. However, it is uncertain whether this process results in elevated levels of IL‐5, eotaxins, and other eosinophil‐associated molecules within the spinal cord lesions. Further studies are necessary to clarify the role of eosinophils in CNS lesions of patients with MOGAD, particularly in cases of post‐vaccination demyelination.

This case study has several limitations. First, data on CSF levels of IL‐5, eotaxins, and other eosinophil‐associated molecules are unavailable because CSF samples from our patient were not preserved. Second, there is no evidence that MOG‐IgG was negative prior to influenza vaccination, as neither serum nor CSF samples were collected before vaccination, although MOG‐IgG production was presumably induced by vaccination based on the timing between vaccination and symptom onset.

In conclusion, the presence of CSF eosinophils in our case supports the recent speculation that perivascular infiltration of eosinophils can occur in demyelinating lesions among patients with MOGAD. This eosinophil infiltration may be attributed to increased levels of eosinophil‐associated cytokines and chemokines within the CNS lesions. However, it remains undetermined whether immunological responses triggered by vaccination can promote the production of these mediators in such lesions, and further studies are needed.

## Funding

This work was supported by an intramural fund from the National Centre of Neurology and Psychiatry, Grant/Award Number: 6‐8.

## Consent

Written informed consent was obtained from the patient for the publication of this case report.

## Conflicts of Interest

The authors declare no conflicts of interest.

## Data Availability

The data supporting the findings of this study are available from the corresponding author upon reasonable request.
